# Methamphetamine-induced changes in myocardial gene transcription are sex-dependent

**DOI:** 10.1186/s12864-021-07561-x

**Published:** 2021-04-12

**Authors:** Hasitha Chavva, Daniel A. Brazeau, James Denvir, Donald A. Primerano, Jun Fan, Sarah L. Seeley, Boyd R. Rorabaugh

**Affiliations:** 1 Department of Pharmaceutical Science, Marshall University School of Pharmacy, 1 John Marshall Drive, Huntington, WV 25755 USA; 2 Department of Pharmacy Practice, Administration, and Research, Marshall University School of Pharmacy, 1 John Marshall Drive, Huntington, WV 25755 USA; 3 Department of Biomedical Science, Marshall University School of Medicine, 1 John Marshall Drive, Huntington, WV 25755 USA; 4 Department of Pharmaceutical and Biomedical Sciences, Ohio Northern University College of Pharmacy, 525 South Main Street, Ada, OH 45810 USA

**Keywords:** Methamphetamine, Heart, Transcriptome, Sex differences, Circadian clock, Drug abuse

## Abstract

**Background:**

Prior work demonstrated that female rats (but not their male littermates) exposed to methamphetamine become hypersensitive to myocardial ischemic injury. Importantly, this sex-dependent effect persists following 30 days of subsequent abstinence from the drug, suggesting that it may be mediated by long term changes in gene expression that are not rapidly reversed following discontinuation of methamphetamine use. The goal of the present study was to determine whether methamphetamine induces sex-dependent changes in myocardial gene expression and whether these changes persist following subsequent abstinence from methamphetamine.

**Results:**

Methamphetamine induced changes in the myocardial transcriptome were significantly greater in female hearts than male hearts both in terms of the number of genes affected and the magnitude of the changes. The largest changes in female hearts involved genes that regulate the circadian clock (*Dbp, Per3, Per2, BMal1*, and *Npas2*) which are known to impact myocardial ischemic injury. These genes were unaffected by methamphetamine in male hearts. All changes in gene expression identified at day 11 returned to baseline by day 30.

**Conclusions:**

These data demonstrate that female rats are more sensitive than males to methamphetamine-induced changes in the myocardial transcriptome and that methamphetamine does not induce changes in myocardial transcription that persist long term after exposure to the drug has been discontinued.

**Supplementary Information:**

The online version contains supplementary material available at 10.1186/s12864-021-07561-x.

## Background

Methamphetamine is one of the most commonly used drugs of abuse in the United States and world-wide. The 2018 National Survey on Drug Use and Health indicated that approximately 5% of US residents 12 years of age or older have used methamphetamine at least once during their lifetime [1]. The Centers for Disease Control reported that the age-adjusted rate of overdose deaths involving amphetamine and amphetamine derivatives increased more than 4-fold between 2012 (0.9 deaths / 100,000 population) and 2018 (3.9 deaths / 100,000 population) [2]. In some areas of the United States, the number of overdose deaths involving methamphetamine exceeds the number of deaths involving opioids [3].

Methamphetamine use increases the risk of cardiovascular disorders including cardiomyopathy [4, 5], atherosclerosis [6], myocardial infarction [7–10], fibrosis [6], cardiac arrhythmias [11, 12], and stroke [11, 13]. We previously reported that adult female rats treated with methamphetamine develop myocardial hypersensitivity to ischemic injury [14]. In contrast, methamphetamine had no effect on myocardial sensitivity to ischemia in their male littermates. Importantly, this sex-dependent effect persisted following a 1-month period of subsequent abstinence from methamphetamine, indicating that methamphetamine induces sex-dependent effects in the heart that persist even after exposure to the drug has been discontinued. A similar sex-dependent effect occurred in adult rats that were exposed to methamphetamine during the prenatal period. Female rats that were prenatally exposed to methamphetamine developed myocardial hypersensitivity to ischemia during adulthood. However, their male littermates were unaffected [15]. These studies suggest that methamphetamine (during either the prenatal period or during early adulthood) may induce changes in gene expression that are sex-dependent and that persist after methamphetamine use has been discontinued.

Other investigators have reported that methamphetamine induces changes in gene expression in the nucleus accumbens [16, 17], frontal cortex [18, 19], dorsal striatum [20], and hippocampus [19] that persist following 3–6 weeks of subsequent abstinence from the drug. However, it is unknown whether methamphetamine induces long term changes in gene expression in the heart. The goal of the present study was to determine whether methamphetamine exposure during early adulthood induces sex-dependent changes in gene expression that may underlie the drug’s ability to selectively hypersensitize the female heart to ischemic injury. Based on our prior studies, we hypothesized that methamphetamine produces sex-dependent changes in myocardial gene expression that persist following a 1-month period of subsequent abstinence from the drug.

## Results

### Impact of methamphetamine on body weight and heart weight

Animals used in this study were 8 weeks of age. Body weights on the first day of saline or methamphetamine (5 mg/kg) injections were similar for male rats treated with either saline (324 ± 12 g) or methamphetamine (343 ± 7 g). Three-way ANOVA indicated significant effects of sex (males gained more weight than females), time (rats gained more weight over 10 days compared to 40 days), and an interaction between time and methamphetamine (effect of methamphetamine was different following 10 days of treatment compared to 10 days of treatment followed by 30 days abstinence), but there was no significant effect of methamphetamine on weight gain (Table 1). Weight gain in females showed the same pattern. Starting weights were similar on the first day of saline (213 ± 9 g) or methamphetamine (214 ± 6) treatment. Methamphetamine-injected females gained nominally less weight than saline-injected females over the course of the 10-day treatment period, but this effect was not statistically significant (Table 1).

**Table 1:** Heart weight and heart weight / body weight ratio of rats following 10 days of saline / methamphetamine injections or 10 days saline / methamphetamine injections followed by 30 days of subsequent abstinence. Data represent the mean ± SEM of 6 animals

	***N***	Heart weight (g)	Heart weight / body weight ratio (mg/g)	Weight Gain (g)
**Group 1: Male 10 days saline**	6	1.5 ± 0.05	3.9 ± 0.2	46 ± 1
**Group 2: Male 10 days methamphetamine**	6	1.6 ± 0.06	4.1 ± 0.1	37 ± 2
**Group 3: Female 10 days saline**	6	0.95 ± 0.04^a^	4.5 ± 0.1	19 ± 2^a^
**Group 4: Female 10 days methamphetamine**	6	0.95 ± 0.02^b^	4.5 ± 0.1	11 ± 2^b^
**Group 5: Male 10 days saline + 30 days abstinence**	6	1.7 ± 0.06^a^	3.8 ± 0.1	104 ± 6^a^
**Group 6: Male 10 days methamphetamine + 30 days abstinence**	6	1.7 ± 0.06	3.6 ± 0.2	110 ± 8^g^
**Group 7: Female 10 days saline + 30 days abstinence**	6	1.2 ± 0.03^c^	4.0 ± 0.2^e^	46 ± 2^c^
**Group 8: Female 10 days methamphetamine + 30 days abstinence**	6	1.1 ± 0.02^d^	3.8 ± 0.1^f^	44 ± 7^d^

Three-way ANOVA indicated significant effects of sex [F = 353 (1, 40) *p* < 0.0001] and time (10 days treatment vs 10 days treatment + 30 days abstinence) [F = 42 (1, 40), *p* < 0.0001] on heart weight. There were also significant effects of sex [F = 12 (1, 40), *p* = 0.001] and time [F = 24 (1, 40), *p* < 0.0001] on heart weight / body weight ratio and significant effects of sex [F = 311 (1, 64), *p* < 0.0001] and time [F = 368 (1, 64), p < 0.0001] on weight gain. There was also a significant interaction between methamphetamine and time [F = 4.5 (1, 64), *p* < 0.05] on weight gain. However, methamphetamine alone had no significant effect on heart weight, heart weight / body weight ratio, or weight gain. ^a^ *p* > 0.0001 compared to male 10 day saline; ^b^ p < 0.0001 compared to male 10 day methamphetamine; ^c^ *p* < 0.0001 compared to male 10 days saline + 30 days abstinence; ^d^ *p* < 0.0001 compared to female 10 days methamphetamine + 30 days abstinence; ^e^ *p* < 0.0001 compared to female 10 day saline; ^f^ *p* < 0.0001 compared to female 10 days methamphetamine; ^g^ *p* < 0.05 compared to male 10 days saline

Heart weights and heart weight / body weight ratios were not significantly impacted by methamphetamine in male or female rats following either 10 days of saline or methamphetamine treatment or after a subsequent 30-day period of abstinence from saline or methamphetamine (Table 1). There was no evidence of hypertrophy or other gross anatomical changes in the hearts.

### Sex-dependent effects of methamphetamine on myocardial gene expression following 10 days of methamphetamine or saline injections

Principal component analysis of RNA sequencing data (using the 500 most variable genes) and sample clustering based on sample-sample distances were performed on a regularized log-transformation of the count data. One sample (from a female treated with saline without subsequent abstinence) was observed as an extreme outlier (Supplemental Fig. 1). This library was excluded from subsequent assessment of differential gene expression.

**Fig. 1:**
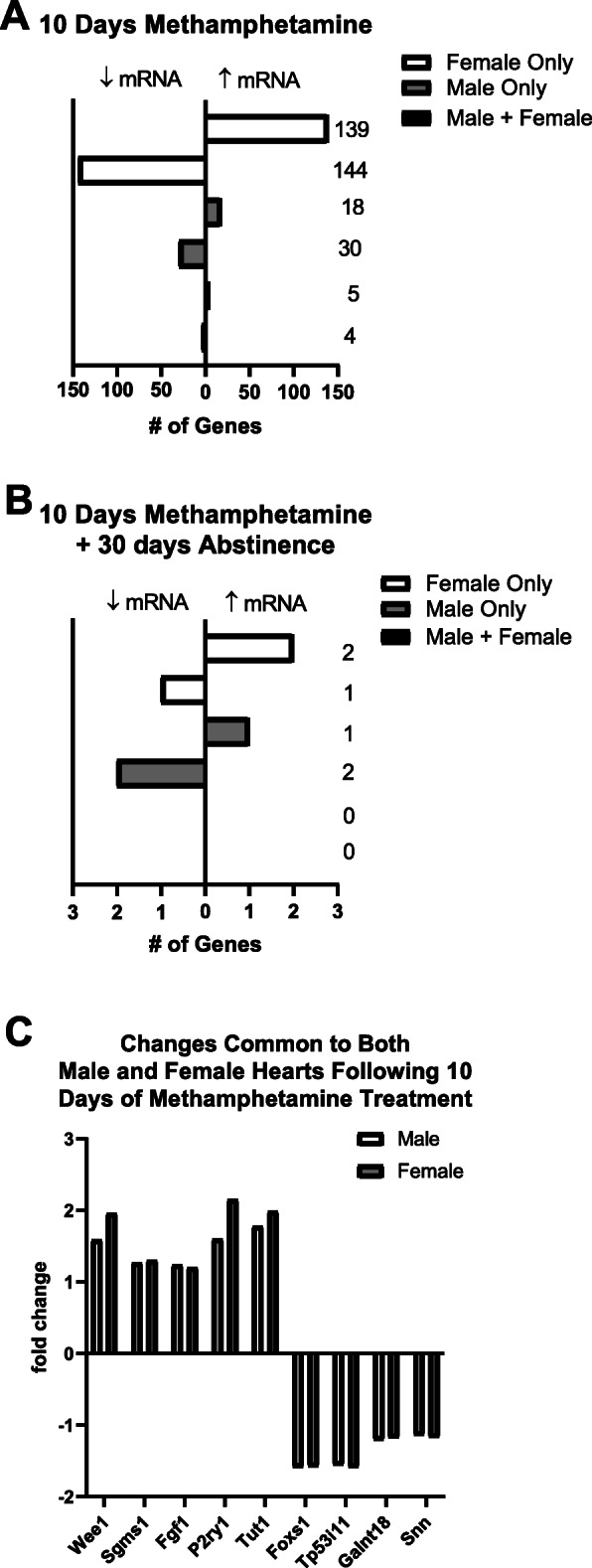
Methamphetamine-induced changes in gene expression are more prevalent in female hearts than in male hearts. RNA sequencing identified changes in the transcription of 346 genes. Changes in 340 genes were identified 24 h after the last methamphetamine injection **a**. Only 6 changes were identified following 30 days of subsequent abstinence **b**. Most (97%) methamphetamine-induced changes in gene transcription were sex dependent with the majority (82%) occurring exclusively in female hearts **a**. Methamphetamine induced changes in only 9 transcripts that were common to both male and female hearts **a, c**

Methamphetamine induced significant changes (false discovery rate < 0.10) in the transcription of 346 genes. This included 340 changes identified 24 h after the last methamphetamine injection (Fig. 1a) and 6 changes following 30 days of subsequent abstinence (Fig. 1b). Most (82%) methamphetamine-induced changes in gene expression occurred exclusively in female hearts [283 changes exclusively in females after 10 days of methamphetamine (Fig. 1a) plus 3 changes following 30 days of subsequent abstinence (Fig. 1b)], and most changes (98%; 340 out of 346 total changes) were identified 24 h after the last injection (Fig. 1a). Only 6 (2% of all changes) methamphetamine-induced changes in gene expression were identified 30 days after the last injection (Fig. 1b). Most methamphetamine-induced changes that were common to both male and female hearts were less than 2-fold in magnitude (Fig. 1c) and had no distinct functional commonalities with one another. Changes in gene transcription in female hearts that were 2-fold or greater in magnitude following 10 days of methamphetamine treatment are shown in Table 2. There were no changes in transcription greater than 2-fold in magnitude in male hearts. The lists of all changes identified exclusively in female hearts or exclusively in male hearts are shown in Supplemental Table 1 and Supplemental Table 2, respectively. All sequencing data have been submitted to NCBI GEO and are available via accession number GSE158655.

**Table 2:** Changes in gene expression (2-fold or greater) in female hearts following 10 days methamphetamine treatment. FPKM values represent the mean ± SEM of 5 saline-treated and 6 methamphetamine-treated animals

Ensembl Identification	symbol	Gene Name	Saline FPKM	Meth FPKM	Effect (Fold up/ down)	Padj
ENSRNOG00000021027	Dbp*	D-site binding protein	8.2 ± 3.7	76.4 ± 31	9.3 Up	2.1 E-05
ENSRNOG00000050675	Myl4	Myosin Light Chain 4	4.6 ± 1.9	37.4 ± 17	8.2 Down	0.00024
ENSRNOG00000018413	Per3*	Period Circadian Regulator 3	2.1 ± 0.9	17.3 ± 7.1	8.2 Up	9.3 E-06
ENSRNOG00000020254	Per2*	Period Circadian Regulator 2	3.3 ± 1.5	20.3 ± 8.3	6.1 Up	4.0 E-05
ENSRNOG00000014448	Bmal1 / Arntl*	Aryl hydrocarbon receptor nuclear translocator-like protein 1	21.9 ± 9.8	3.9 ± 1.6	5.6 Down	0.019
ENSRNOG00000013408	Npas2*	Neuronal PAS domain protein 2	7.3 ± 3.3	1.7 ± 0.7	4.3 Down	0.0013
ENSRNOG00000000633	Rhobtb1	Rho Related BTB Domain Containing 1	8.7 ± 3.9	37.2 ± 15.2	4.3 Up	5.9E-06
ENSRNOG00000006033	Spon2	Spondin 2	8.3 ± 3.7	2 ± 0.8	4.2 Down	7.5E-05
ENSRNOG00000029980	Zbtb16	Zinc Finger and BTB Domain containing 16	4.8 ± 2.1	18.7 ± 7.6	3.9 Up	0.00033
ENSRNOG00000019383	Tef	Thyrotroph embryonic factor	18 ± 8	62.4 ± 25.5	3.5 Up	8.8E-06
ENSRNOG00000020525	Col5a3	Collagen type V Alpha 3 chain	13.5 ± 6	4 ± 1.6	3.4 Down	5.9E-06
ENSRNOG00000001469	Eln	Elastin	45 ± 20	14 ± 5.7	3.2 Down	2.1E-05
ENSRNOG00000045821	Slc41a3	Solute carrier Family 41 Member 3	57.2 ± 25.6	19.3 ± 7.9	2.963 Down	0.00017
ENSRNOG00000046912	Nr1d2	Nuclear receptor subfamily 1, group D, member 2	11.1 ± 5	30.5 ± 12.4	2.7 Up	0.00017
ENSRNOG00000010947	Mmp14	Matrix metallopeptidase 14	22.9 ± 10.2	8.4 ± 3.4	2.7 Down	1.2E-05
ENSRNOG00000003515	Ephx1	Epoxide hydrolase 1	7.5 ± 3.3	20.2 ± 8.3	2.7 Up	1.5E-05
ENSRNOG00000060511	NA	Neuraminidase	8.8 ± 3.9	23.3 ± 9.5	2.6 Up	2.9E-05
ENSRNOG00000049422	Fcgr2a	Fc fragment of IgG receptor II a	6.5 ± 2.9	2.5 ± 1	2.6 Down	0.0026
ENSRNOG00000000521	Cdkn1a	Cyclin dependent kinase inhibitor 1A	31.8 ± 14.2	12.2 ± 5	2.6 Down	0.00089
ENSRNOG00000046663	Fcgr2a	Fc fragment of IgG receptor II a	11.6 ± 5.2	4.5 ± 1.8	2.6 Down	0.0015
ENSRNOG00000056153	Fam46b	Family with sequence similarity 46, member B	4.8 ± 2.1	11.9 ± 4.9	2.5 Up	0.018
ENSRNOG00000000885	LOC100362819		8.9 ± 4	3.6 ± 1.5	2.5 Down	0.00024
ENSRNOG00000001414	Serpin E1	Endothelial plasminogen activator inhibitor-1	7.2 ± 3.2	17.3 ± 7.1	2.4 Up	5.5E-05
ENSRNOG00000029810	Tspan4	Tetraspanin-4	12.1 ± 5.4	29.2 ± 11.9	2.4Up	0.00053
ENSRNOG00000055564	RGD1564664		9.5 ± 4.2	4.1 ± 1.7	2.3 Down	0.0019
ENSRNOG00000017716	Ucp3	Mitochondrial uncoupling protein 3	10.5 ± 4.7	4.6 ± 1.9	2.3 Down	0.014
ENSRNOG00000049324	LOC100911766		5.5 ± 2.5	12.4 ± 5.1	2.3 Up	0.0005
ENSRNOG00000008680	Loxl1	Lysyl oxidase homolog 1	14.9 ± 6.7	6.7 ± 2.7	2.2 Down	8.6E-05
ENSRNOG00000058500	LOC103694908		10.7 ± 4.8	4.9 ± 2.0	2.2 Down	0.015
ENSRNOG00000000906	Medag	Mesenteric estrogen dependent adipogenesis	20 ± 9	9.3 ± 3.8	2.2 Down	7.3E-05
ENSRNOG00000011800	F3	Platelet tissue factor	5.5 ± 2.5	12 ± 5	2.2 Up	0.001
ENSRNOG00000004699	Fibin	Fin Bud Initiation Factor Homolog	9.4 ± 4.2	4.4 ± 1.8	2.1 Down	0.0007
ENSRNOG00000003228	Mid1ip1	MID 1 Interacting Protein 1	31.6 ± 14.1	67.7 ± 27.6	2.1 Up	7.6E-06
ENSRNOG00000006663	Usp2	Ubiquitin Specific Peptidase 2	14.2 ± 6.3	30.1 ± 12.3	2.1 Up	0.015
ENSRNOG00000016573	Dgat2	Diacylglycerol O-Acyltransferase 2	5.8 ± 2.6	12.1 ± 5	2.1 Up	0.0019
ENSRNOG00000005271	Rapgef5	Rap guanine nucleotide exchange factor 5	8.8 ± 3.9	17.6 ± 7.2	2.0 Up	1.2E-05

Effects labeled “up” or “down” indicate methamphetamine-induced increases or decreases in FPKM values of methamphetamine-treated rats compared to saline treated rats. * indicates genes involved in regulating the circadian clock

### Methamphetamine induced changes in gene transcription are larger in magnitude in female hearts compared to male hearts

**Fig. 2:**
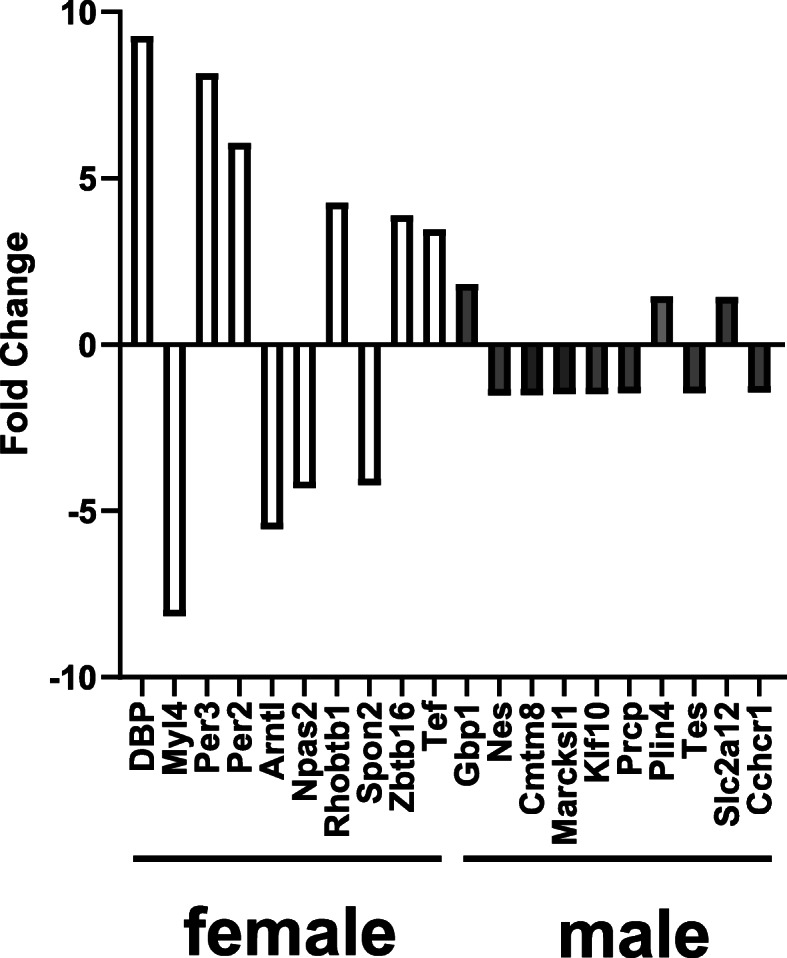
Methamphetamine induced larger changes in gene transcription in female hearts compared to male hearts. Methamphetamine-induced changes in gene transcription were ranked according to their magnitude. The top 10 genes (in terms of the largest methamphetamine-induced changes) in male and female hearts were identified. The magnitude of methamphetamine-induced change in gene transcription was significantly (*p* < 0.0001) greater in female hearts (5.7 ± 0.7-fold) than in male hearts (1.5 ± 0.04-fold)

The top 10 changes in gene transcription (in terms of the magnitude of changes to individual genes) averaged 5.7 ± 0.7-fold in female hearts compared to a 1.5 ± 0.04-fold change in male hearts (Fig. 2). Thus, methamphetamine had a greater impact on myocardial gene expression in female hearts than in males, both in terms of the number of genes that were upregulated / downregulated (Fig. 1) and the magnitude (Fig. 2) of the changes.

### Global expression profiling analysis

Global expression profiling of differentially expressed genes identified pathways and functions that were overrepresented following 10 days of methamphetamine treatment. The top 20 pathways and functions (ordered by false discovery rate) altered by methamphetamine in male and female hearts are shown in Table 3. Supplemental Table 3 and Supplemental Table 4 show the full list of pathways with FDR < 0.05 in female and male hearts, respectively. We observe that 6 of the top 20 functions and pathways for female hearts are associated with circadian rhythms, whereas these functions are absent in the comparison for the male hearts.

**Table 3:** Top 20 functions and pathways (ranked by False Discovery Rate) associated with differentially expressed genes in female (**A**) or male (**B**) hearts following 10 days of injection with either methamphetamine or saline

# of Enriched Genes	Category	Description	FDR value
**A. Female Hearts**			
12	NetworkNeighborAL	Circadian rhythm, and PAR basic leucine zipper protein	3.03E-10
11	NetworkNeighborAL	Circadian rhythm, and PAR basic leucine zipper protein	3.18E-10
17	Reference publications	Analysis of gene regulatory networks in the mammalian circadian rhythm [21]	7.19E-09
73	GO Process	negative regulation of cellular process	9.21E-09
78	GO Process	negative regulation of biological process	9.21E-09
62	GO Process	regulation of biosynthetic process	9.21E-09
14	NetworkNeighborAL	mixed, incl. Circadian rhythm, and Basic region leucine zipper	3.32E-08
42	GO Process	response to abiotic stimulus	4.32E-08
13	NetworkNeighborAL	mixed, incl. Circadian rhythm, and Basic region leucine zipper	5.19E-08
107	GO Process	response to stimulus	6.70E-08
78	GO Process	response to chemical	6.70E-08
75	GO Process	regulation of primary metabolic process	1.15E-07
94	GO Function	protein binding	1.32E-07
152	GO Process	cellular process	1.32E-07
57	GO Process	regulation of cellular biosynthetic process	1.39E-07
79	GO Process	regulation of metabolic process	1.39E-07
39	GO Process	positive regulation of biosynthetic process	1.39E-07
8	NetworkNeighborAL	Circadian rhythm	1.57E-07
75	GO Process	regulation of cellular metabolic process	1.89E-07
116	GO Process	regulation of cellular process	2.09E-07
**B. Male Hearts**			
3	Reactome Pathways	Activation of C3 and C5	0.0035
4	UniProt Keywords	Multifunctional enzyme	0.0043
23	UniProt Keywords	Acetylation	0.0043
38	UniProt Keywords	Phosphoprotein	0.0053
6	Reference publications	Fibrosis and progression of autosomal dominant polycystic kidney disease (ADPKD) [22]	0.0098
16	GO Function	carbohydrate derivative binding	0.0147
20	GO Function	anion binding	0.0147
30	GO Function	ion binding	0.0147
10	GO Function	cytoskeletal protein binding	0.0147
32	GO Function	protein binding	0.0147
47	GO Function	binding	0.0147
6	Reference publications	Recent highlights on bone stem cells: a report from Bone Stem Cells 2009, and not only [23].	0...0167
6	UniProt Keywords	Actin-binding	0.0176
4	Reference publications	Transitional Remodeling of the Hepatic Extracellular Matrix in Alcohol-Induced Liver Injury [24]	0.0187
4	Reference publications	Influence of culture conditions and extracellular matrix alignment on human mesenchymal stem cells invasion into decellularized engineered tissues [25]	0.0187
5	Reference publications	(2014) Osteopontin deletion prevents the development of obesity and hepatic steatosis via impaired adipose tissue matrix remodeling and reduced inflammation and fibrosis in adipose tissue and liver in mice [26]	0.0187
17	UniProt Keywords	Nucleotide-binding	0.0214
17	GO Function	small molecule binding	0.0238
6	GO Function	actin binding	0.0238
4	GO Function	actin filament binding	0.0275

### Changes in gene expression following 10 days of methamphetamine injections followed by 30 days of subsequent abstinence

All methamphetamine-induced changes in gene transcription observed following 10 days of methamphetamine treatment returned to baseline following 30 days of subsequent abstinence from the drug, indicating that methamphetamine does not induce changes in cardiac gene expression that persist long-term after the drug has been discontinued. Treating male and female rats with methamphetamine followed by 30 days of subsequent abstinence resulted in changes in mRNA transcripts encoding 6 genes (3 in male hearts and 3 in female hearts) compared to control rats that were treated with saline for 10 days prior to 30 days of subsequent abstinence (Fig. 1b; Table 4). These genes were not identified as significantly different in hearts collected immediately after 10 days of methamphetamine exposure. Global expression profiling analysis was not performed in these hearts because of the small number of changes in gene transcription that were identified (3 changes in male and 3 changes in female) following the period of abstinence.

**Table 4:** Changes in myocardial gene expression in rats treated with saline or methamphetamine (5 mg/kg) for 10 days followed by 1 month of abstinence

		Saline FPKM	Meth FPKM	Effect (Fold Up/Down)	P_**adj**_
**Female**	Ptprc ENSRNOG00000000655	4.5 ± 0.1	7.6 ± 1.6	1.7 Up	0.007
	Hmgb2 ENSRNOG00000013167	17 ± 1	27 ± 5	1.6 Up	0.007
	GCAT ENSRNOG00000055408	17 ± 0.7	14 ± 0.9	1.3 Down	0.02
**Male**	Nr1d1 ENSRNOG00000009329	13 ± 2	37 ± 6	2.9 Up	0.01
	Cry1 ENSRNOG00000006622	5.9 ± 0.4	3.2 ± 0.5	1.8 Down	0.02
	Nfil3 ENSRNOG00000011668	14 ± 0.6	7.9 ± 0.8	1.8 Down	0.02

FPKM values represent the mean ± SEM of 5–6 animals

### Methamphetamine sex-dependently alters transcription of genes that regulate the circadian clock

**Fig. 3:**
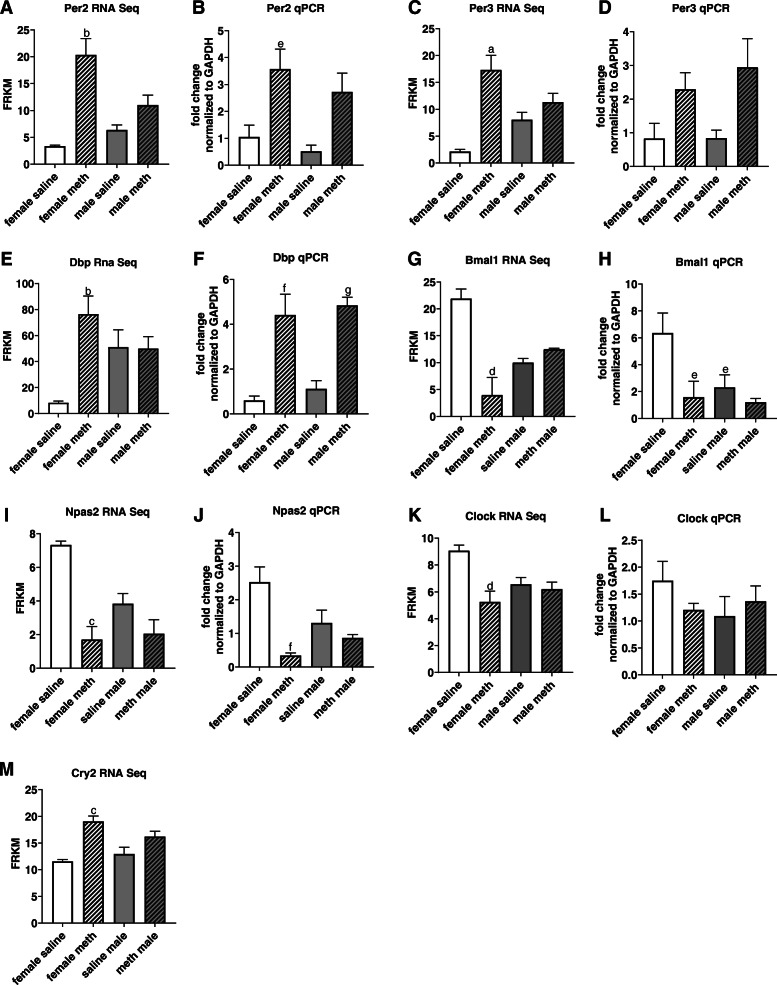
Methamphetamine selectively regulates transcription of circadian clock-related genes in female hearts. Methamphetamine-induced changes in genes that regulate the circadian rhythm were identified by RNA sequencing and qPCR. RNA sequencing identified significant effects of methamphetamine on the number of transcripts encoding Per2 **a**, Per3 **c**, Dbp **e**, Bmal1 **g**, Npas2 **i**, Clock **k**, and Cry2 **m**. Two-way ANOVA of qPCR data indicated significant effects of methamphetamine on the number of transcripts encoding Per2 [F = 17 [1, 20], *p* < 0.0005] **b**, Dbp [F = 47 [1, 20], *p* < 0.0001] **f**, BmalI [F = 51 [1, 20], *p* < 0.05] **h**, and Npas2 [F = 19 [1, 19], *p* < 0.0005] **j**. There was also a significant interaction between sex and methamphetamine on Npas transcription [F = 8 [1, 19], *p* < 0.01] **j**. ^a^ indicates p_adj_ < 0.0001 compared to hearts from saline treated females. ^b^ indicates p_adj_ < 0.00005 compared to hearts from saline treated females. ^c^ indicates p_adj_ < 0.0005 compared to hearts from saline treated females. ^d^ indicates p_adj_ < 0.001 compared to hearts from saline treated females. ^e^ indicates *p* < 0.05 vs female saline; ^f^ indicates *p* < 0.0005 vs female saline; ^g^ indicates *p* < 0.0005 vs male saline. Data represent the mean ± SEM of 5–6 animals

Methamphetamine-induced changes in gene expression in female hearts (following 10 days of saline or methamphetamine treatment) were ranked according to the magnitude of the methamphetamine-induced effect (Table 2). Notably, 5 of the top 6 changes in gene expression (in terms of the magnitude of changes) in hearts from female rats involved genes that regulate the circadian clock. mRNA transcripts encoding Per2 (Fig. 3a), Per3 (Fig. 3c), and Dbp (Fig. 3e) increased at least 6-fold following 10 days of methamphetamine treatment, while BmalI (Fig. 3g and Npas2 (Fig. 3i) demonstrated 4.3 and 5.5-fold decreases in mRNA transcripts, respectively. RNA sequencing identified smaller (but statistically significant) changes in the transcription of additional circadian clock-related genes in female hearts including CLOCK (Fig. 3k), and Cry2 (Fig. 3m). RNA sequencing identified no methamphetamine-induced changes in the transcription of these genes in male hearts (Fig. 3 panels A, C, E, G, I, K, and M). Furthermore, these sex-dependent changes in gene transcription were reversible, as none of the changes persisted in female hearts following 30 days of abstinence from the drug.

Quantitative PCR was used to confirm changes in the expression of selected genes that regulate the circadian clock. Results from qPCR mirrored those from RNA sequencing for transcripts encoding Per 2 (Fig. 3a-b), BMAL1 (Fig. 3g-h), and NPAS2 (Fig. 3i-j). qPCR data were also similar to those of RNA sequencing for Per3 (Fig. 3c-d) and CLOCK (Fig. 3k-l), but these changes only reached statistical significance when measured by RNA sequencing. Data from RNA sequencing and qPCR differed for Dbp in that RNA sequencing identified a methamphetamine-induced increase in Dbp transcripts only in female hearts (Fig. 3e), while qPCR identified this change in both males and females (Fig. 3f). We were unable to assess Cry2 transcripts by qPCR.

### Western blot analysis of proteins that regulate the circadian clock

**Fig. 4:**
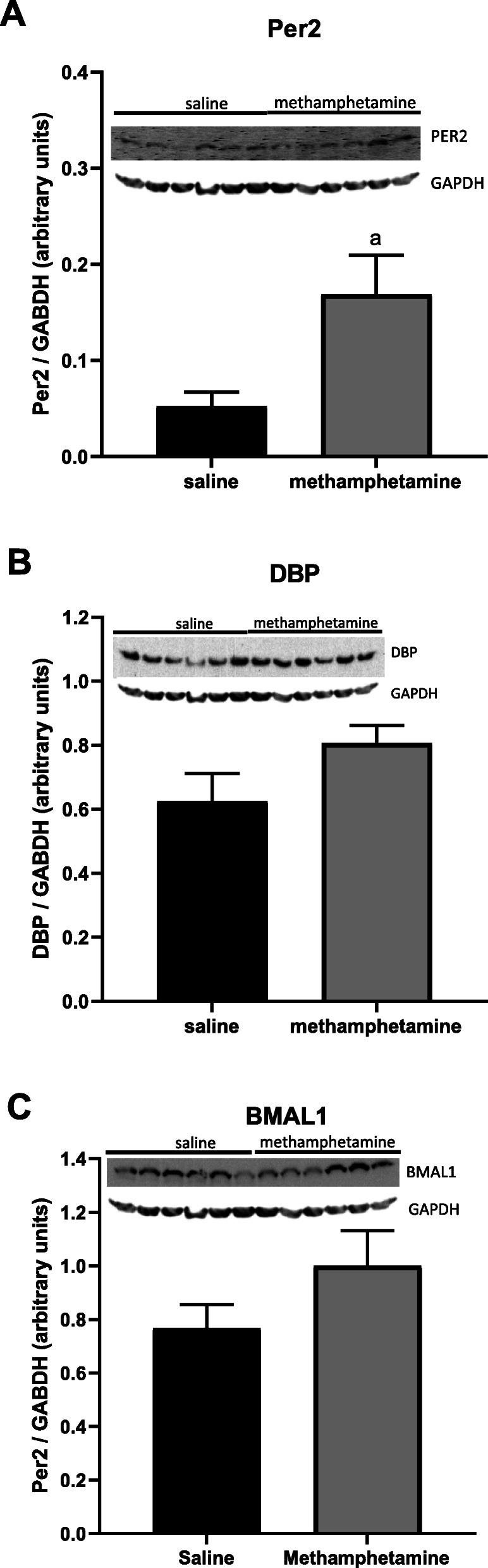
Expression of Per2, DBP, and BMAL1 in female hearts following 10 days of methamphetamine treatment. Female rats were treated with saline or methamphetamine for 10 days. Hearts were isolated and flash frozen on day 11. Expression of Per2 **a**, DBP **b**, and BMAL1 **c** in ventricular tissue was measured by western blot. Values in histograms represent the mean ± SEM of 6 hearts for each group. ^a^ indicates *p* < 0.05. The full-length blots for each protein are shown in Supplemental Fig. 2

Consistent with RNA sequencing and qPCR analyses, western blots indicated that Per2 was significantly upregulated at the protein level in hearts from methamphetamine-treated female rats (Fig. 4a). Dbp (Fig. 4b) and BMALI **(**Fig. 4c) expression were nominally increased at the protein level, but these changes did not reach statistical significance.

## Discussion

The primary findings of this study are that 1) female hearts are more susceptible than male hearts to methamphetamine-induced changes in gene transcription; 2) methamphetamine does not induce long-lasting changes in myocardial gene transcription that persist following 1 month of subsequent abstinence from the drug; and 3) methamphetamine induces sex-dependent changes in the transcription of genes that regulate the circadian clock in the heart.

This work was prompted by our previous finding that methamphetamine treatment for 10 days causes female rats (but not their male siblings) to develop myocardial hypersensitivity to ischemic injury [14]. Importantly, this methamphetamine-induced effect persisted in female hearts following 1 month of subsequent abstinence from the drug, suggesting that it might result from long term changes in cardiac gene expression that are not rapidly reversed when methamphetamine exposure is discontinued. We anticipated that the identification of methamphetamine-induced changes in myocardial gene expression that are both sex dependent (occurring only in females) and that persist following a period of subsequent abstinence from the drug may provide a mechanistic basis for our observations regarding the impact of methamphetamine on the ischemic heart. Contrary to this hypothesis, our findings indicate that methamphetamine does not induce changes in myocardial gene transcription that persist long term after the drug has been discontinued.

Interactions between the period genes, BmalI, Npas2, Dbp, Clock, cryptochromes, and other genes that regulate circadian function are well characterized and have been the subject of recent reviews [27, 28]. The ability of methamphetamine to alter the expression of genes that regulate circadian rhythm in the hippocampus, striatum, and other regions of the brain is well established [29–33]. However, this is the first study that we are aware of to demonstrate that methamphetamine alters the myocardial transcription of clock-related genes (*Per2, Per3, Dbp, Clock, Bmal I, Cry2, Npas2*) in the heart and that this occurs in a sex-dependent manner. The circadian clock plays an important role in regulating diurnal changes in cardiac metabolism, heart rate, and blood pressure [28, 34], and there is evidence from both animal models [35, 36] and human studies [37–39] [40, 41] that disruption of the circadian clock adversely impacts the development of cardiovascular disease [27, 42, 43] and susceptibility to myocardial infarction [36, 44]. Thus, the observation that 10 days of methamphetamine treatment alters the transcription of circadian clock genes and also causes female hearts to become hypersensitive to ischemic injury [14] is consistent with the work of other investigators. However, our findings do not provide an explanation for the observation that these animals remain hypersensitive to ischemia after a 1-month period of subsequent abstinence when transcription of these genes is no longer altered by methamphetamine. Our data do not rule out the possibility that methamphetamine induces epigenetic changes that serve as a “memory” of methamphetamine exposure and subsequently influence transcriptional changes induced by an ischemic insult. Further work is needed to determine whether methamphetamine induces epigenetic changes that alter transcriptional responses triggered by ischemia or other forms of cardiac stress.

Female hearts were significantly more sensitive than male hearts to methamphetamine-induced changes in gene expression, both in terms of the number of genes effected and the magnitude of the methamphetamine-induced changes. This might result from the fact that the rate of clearance of methamphetamine is lower in female rats than in male rats, resulting in females having a greater exposure (in terms of area under the concentration – time curve) than males given an equal dose of the drug [45]. Methamphetamine has been reported to disrupt the hypothalamic-pituitary-ovarian axis in females [46]. Thus, this sex difference could alternatively be secondary to changes in function of the hypothalamic- pituitary - ovarian axis, disruption of the cardioprotective effects of estrogen, or to sex differences in the brain’s response to methamphetamine rather than a direct effect of methamphetamine on the heart. It should be noted that not all methamphetamine-induced cardiac effects occur exclusively in female hearts. Some investigators have reported that males are more susceptible than females to methamphetamine-induced cardiomyopathy [47, 48]. Additional work is needed to understand the mechanism by which methamphetamine induces sex-dependent effects in the myocardium.

The finding that the female heart is more sensitive than the male heart to methamphetamine-induced changes in gene expression is consistent with previously reported cardiac sex-differences. Sexual dimorphism in rodent models of cardiovascular health and disease was recently the topic of an extensive review [49]. Baseline sex differences in the activity of ion channels [50], cardiac mitochondrial metabolism [51], cardiac expression of calcium handling proteins [52–54], and sex differences in the concentration of norepinephrine in myocardial tissue [55, 56] have been reported in healthy rodents. Furthermore, the cardioprotective benefit of estrogen is well established [57]. Male hearts are more sensitive than female hearts to myocardial ischemic injury [15, 58–60]. Male rodents are also reported to have more maladaptive cardiac remodeling, poorer recovery of ventricular function, and lower survival rates than females following a myocardial infarction [61, 62]. Sex-differences in the cardiac response to pressure overload, volume overload, and isoproterenol-induced hypertrophy have also been reported [49]. Thus, sex-dependent differences in both cardiac physiology and pathophysiology are well established. Our finding that the female cardiac transcriptome is more sensitive than the male transcriptome to the effects of methamphetamine extends our knowledge of cardiac sex differences.

RNA sequencing identified methamphetamine-induced changes in the number of cardiac transcripts for several circadian rhythm-related genes in the female heart (Fig. 3**)**. Most of these findings were replicated by qPCR (Fig. 3**)**. The qPCR data for Per3 and Clock demonstrated a trend in the same direction as the RNA sequencing data, but the methamphetamine-induced effect did not reach statistical significance for Per3 and Clock when measured by qPCR. RNA sequencing identified a methamphetamine-induced increase in the number of Dbp transcripts in female hearts (Fig. 3e) but no change male hearts. In contrast, qPCR found a significant increase in Dbp transcripts in both sexes (Fig. 3f). It is unclear why there is a disparity between these two methods of measuring Dbp transcripts in male hearts.

Genes encoding Per2, BMALI, and DBP are regulated by negative feedback mechanisms in which expression of the protein suppresses transcription of the gene [63]. Expression is also regulated by ubiquitin-dependent mechanisms that regulate rates of protein degradation [64–67]. These mechanisms result in a cyclic pattern of expression over a 24-h time period. Based on the fact that expression of these proteins is tightly controlled by both transcriptional and proteolytic mechanisms, it is not surprising that data from western blotting experiments (Fig. 4) did not precisely mirror the changes observed at the transcript level (Fig. 3). The assessment of circadian clock genes (both at the transcript and protein levels) at only a single time point is a limitation of this study.

The vast majority of changes in transcripts for both male and female hearts were observed immediately following 10 days of methamphetamine treatment (Fig. 1a). However, changes in the transcripts of 6 additional genes (3 in male hearts and 3 in female hearts) were identified following a 30-day period of subsequent abstinence from methamphetamine. Most (5 out of 6) of these changes were less than 2-fold in magnitude (Fig. 1b; Table 4). It is noteworthy that all 3 changes observed in male hearts following 30 days of abstinence involved genes that regulate the circadian rhythm and that no circadian-related genes were altered in female hearts following 30 days of abstinence (Table 4). Previous studies have documented prolonged periods of disrupted sleep patterns in humans who formerly used methamphetamine [68, 69]. Thus, we speculate that sex differences in the expression of these circadian genes might reflect sex-dependent alterations in sleep patterns associated with the discontinuation of methamphetamine. Further work is needed to understand the mechanism and physiological impact of these changes.

## Conclusions

These data provide evidence that the female heart is more susceptible than the male heart (both in terms of the number of genes effected and the magnitude of the changes) to methamphetamine-induced changes in gene transcription. Importantly, 10 days of methamphetamine treatment selectively altered the transcription of genes related to the circadian clock in female hearts. This is consistent with prior studies demonstrating that methamphetamine selectively worsens ischemic injury in female hearts [14] and that disruption of the circadian clock alters the cardiac response to an ischemic insult [40, 70]. Further work is needed to elucidate the role of methamphetamine-induced changes in the circadian clock and changes in gene expression on methamphetamine-induced cardiac disorders.

## Methods

### Animals

Male and female Sprague Dawley rats (8 weeks of age) from an established breeding colony at Ohio Northern University were used for all experiments. The colony originated from rats purchased from Charles River Laboratories (strain code 001). The animals were pair housed in standard cages with free access to food and water and were maintained on a 12 h / 12 h light / dark schedule (lights on at 07:00). All procedures were approved by the Institutional Animal Care and Use Committee and were performed in compliance with the recommendations published in the eighth edition of *The Guide for the Care and Use of Laboratory Animals*.

### Drug treatment

A total of 48 adult male and female rats (8 weeks of age) were divided into 8 experimental groups of 6 animals each (Table 1). Experimental animals were derived from 5 different female breeders. Animals from each litter were divided across all 8 experimental groups to avoid litter-based biasing of the data. All animals received daily (10:00) subcutaneous injections of either methamphetamine (5 mg/ kg/day) or saline for 10 consecutive days. This dose was used based on our previous work demonstrating that methamphetamine treatment produces sex-dependent myocardial hypersensitivity to ischemia [14, 15]. This dose is also commonly used by other investigators to study the effects of methamphetamine-induced neurological and behavioral effects [71–73], and is within the range of methamphetamine doses (on a mg/kg basis) typically used by people in illicit settings [74]. The subchronic duration (10 days) of this treatment was based on previous work demonstrating that repeated exposure of rats to methamphetamine over the course of 10 days worsens cardiac injury induced by myocardial ischemia [14] and also worsens cerebral injury in a rat model of ischemic stroke [75]. All injections were administered by the same individual throughout the 10-day treatment period to ensure that stress-related handling of the animals was equal among groups for the duration of the study.

Rats were anesthetized with a single injection containing sodium pentobarbital (100 mg/kg) and heparin (5 mg/kg) either 24 h after the last saline /methamphetamine injection (Table 1, Groups 1–4) or 30 days after the last saline / methamphetamine injection (Table 1, Groups 5–8). The anesthetized animals were euthanized by opening the chest cavity and removing their hearts as previously described [76]. This method of euthanasia enables the heart to be rapidly removed while it is still beating which helps to minimize blood coagulation in the cardiac tissue. The hearts were quickly mounted on a Langendorff isolated heart system and perfused with Krebs solution for 5 min to flush blood from the tissue. Hearts were immediately flash frozen in liquid nitrogen and stored at − 80 °C. All heart isolations occurred between 09:00 and 11:00 in the morning. Total RNA was subsequently isolated from left ventricular tissue. Methamphetamine-induced changes in the number of mRNA transcripts were subsequently measured by RNA sequencing. A subset of the genes identified from the RNA sequencing data were further examined using Western blot (protein) and quantitative PCR (QPCR).

### RNA sequencing

Total RNA was isolated using Trizol (Thermo Fisher, Waltham, MA) according to the manufacturer’s instructions. RNA was subsequently processed by the Marshall University Genomics Core Facility (Huntington, WV). RNA integrity was confirmed using an Agilent 2100 (Santa Clara, CA) bioanalyzer to confirm that all RNA samples had RNA integrity numbers greater than 8.5. RNA libraries were prepared from 1 μg of total RNA using Illumina (San Diego, CA) TruSeq Stranded mRNA kits. Library quality and insert size were assessed by electrophoresis on Agilent DNA High Sensitivity DNA chips. The average library insert size was ~ 280 bp. Libraries were quantitated using fluorescence-based Qubit dsDNA HS Assay (Thermo Fisher Scientific) in preparation for high throughput sequencing.

Twenty-four purified libraries (derived from experimental groups 1–4) were combined as Pool #1, while the remaining 24 libraries (groups 5–8) were combined as Pool #2. Each pool (6 pM) was clustered and sequenced on an Illumina HiSeq 1500 in 2 × 50 paired end rapid runs. Approximately 33 million reads per library were generated.

RNA sequencing reads were trimmed to remove low-confidence base calls and adapter sequences using Trimmomatic version 0.38 [77]. Read quality was checked using FastQC [78]. Reads were aligned to the rat genome rn06 obtained from Ensembl, using HISAT2 version 2.1.0 [79], and the resulting BAM files were sorted using SAMtools version 1.9 [80]. Aligned reads were then mapped to known transcripts from Ensembl genes version 94 using the R/Bioconductor package Genomic Alignments version 1.16.0 [81]. Differentially-expressed genes were identified using DESeq2 version 1.20.0 [82] with a false discovery rate (Benjamini-Hochberg adjusted *p*-value) less than 0.1 used as a threshold for statistical significance.

RNA-Seq data were analyzed using DESeq2, which incorporates mechanisms for determining whether the data are consistent with the presumed underlying statistical distribution (a negative binomial distribution). For each comparison, genes failing to meet the assumptions of a negative binomial distribution were removed from analysis by the DESeq2 algorithm.

### Global expression profiling analysis

In order to determine pathways and functions globally represented by sets of differentially expressed genes, we generated networks of protein-protein interactions for the protein products corresponding to differentially expressed genes using StringDB [83] and performed an enrichment analysis, identifying networks and pathways overrepresented by these protein products. These analyses were conducted in Cytoscape version 3.7.1 [84] using the StringApp plugin, version 1.4.2 with default parameter settings. Overrepresentation analyses were performed for the comparisons that resulted in at least 50 differentially expressed genes: methamphetamine versus saline in males without abstinence and methamphetamine versus saline in females without abstinence. In order to focus on biological pathways and functions, GO Component terms were filtered from the analysis.

### Quantitative polymerase chain reaction (QPCR)

Total RNA was isolated from 30 mg of left ventricular tissue using the Promega (Madison, WI) SV Total RNA isolation kit according to the manufacturer’s instructions. The RNA was resuspended in 50 μl of nuclease free sterile water and stored at − 80 °C. Total RNA concentrations were determined using a Nanovue spectrophotometer (GE Healthcare, USA). cDNA synthesis was done using 165 ng total RNA using the iScript cDNA synthesis Kit (Bio-Rad, Hercules CA). Quantitative polymerase chain reaction was conducted for each gene of interest using 3 μl of cDNA. Gene expression was quantified by TaqMan™ single gene expression assays (Thermo Fisher Scientific, Foster City CA) using Bio-Rad CFX96 Real-Time PCR Detection system (Bio-Rad Laboratories, Inc. Hercules CA). The thermocycle included an initial uracil-DNA glycosylases (UNG) incubation of 50 °C for 2 min to minimize possible carryover contamination, enzyme activation at 95 °C for 30 s, followed by 40 cycles of PCR (denaturation at 95 °C for 15 s; annealing and extension at 60 °C for 20 s). TAQMAN™ probe FAM detection was assessed at the end of each extension step. Gene expression was assessed for *Per 2* (Period Circadian Regulator 2, Rn01427704_m1), *Per 3* (Period Circadian Regulator 3, Rn00709499_ml), *Clock* (Rn00573120_ml), *Dbp* (D-Box binding protein, Rn01498425_m1), *Bmal 1/Arntl* (Aryl Hydrocarbon Receptor Nuclear Translocator Like, Rn00577590_m1), and *Npas 2* (Neuronal PAS Domain Protein 2, Rn01438223_m1). QPCR for each sample was performed in duplicate, and gene expression was reported as mean Cq values normalized to glyceraldehyde-3-phosphate dehydrogenase (Rn01749022_g1). Gene expression was reported as a fold change relative to the average expression of all samples.

### Western blots

Frozen left ventricular tissue was homogenized with a Polytron in homogenization buffer [50 mM Tris, pH 7.4; 1 mM EDTA; 1% sodium dodecyl sulfate; phosphatase inhibitor cocktail 2 (catalog no. P5726, Sigma); phosphatase inhibitor cocktail 3 (catalog no. P0044, Sigma); and protease inhibitor cocktail (catalog no. P8340, Sigma)] and immediately boiled for 5 min. Homogenates were centrifuged at 4 °C for 10 min at 14,000 rpm. Supernatant protein (30 μg) was separated on a 10% polyacrylamide electrophoresis gel and subsequently transferred to a nitrocellulose membrane. The membrane was blocked with 5% nonfat dry milk and then blotted overnight with antibodies Dbp (Thermo Fisher Catalog # PA5–4045; Waltham, MA), BmalI (Cell Signaling Catalog # 14020; Danvers, MA), or glyceraldehyde-3-phosphate dehydrogenase (Cell Signaling, Danvers, MA; Catalog # 2118). Western blots were quantified by measurement of band densities with ImageJ software. Band intensities of Dbp and BmalI were normalized to those of GAPDH.

### Statistical analysis

Heart weight, gain in body weight, and heart weight / body weight ratio were analyzed by 3 way ANOVA with methamphetamine treatment (saline vs methamphetamine), sex (male vs female), and time (10 days drug treatment vs 10 days drug treatment + 30 days abstinence) as factors. Body weight was analyzed as a repeated measure.

Differentially expressed genes in RNA sequencing experiments were identified using DESeq2 version 1.20.0 [82] with an false discovery rate (Benjamini-Hochberg adjusted *p*-value) less than 0.1 used as a threshold for statistical significance. Statistical analyses of RNA sequencing data were limited to comparisons between hearts from saline and methamphetamine-treated animals of the same sex. Following advice from the Marshall University Genomics and Bioinformatics Core Facility, a sample size of 6 replicates per biological condition and a sequencing depth of 20 million reads per sample was chosen to optimize statistical power.

Quantitative polymerase chain reaction (QPCR) data were analyzed by two-way ANOVA (factors = drug treatment and sex) and Tukey’s posthoc analysis. *P* values ≤0.05 were regarded as statistically significant. Western blots were analyzed by the student’s t test. These statistical analyses were performed using Graphpad Prism (San Diego, CA) software.

## Supplementary Information

**Additional file 1: Supplemental Figure 1.** Principal component analysis plot of all 48 samples using the 500 genes with the most between-sample variance.

**Additional file 2: Supplemental Figure 2.** Full length western blots for BMAL1, DBP, and GAPDH. Proteins from saline and methamphetamine-treated rat hearts were separated on a 10% polyacrylamide gel and blotted onto nitrocellulose membrane. The membrane was cut at the 50 KDa marker (indicated by the dotted line). The top part of the membrane was blotted for BMAL1 (**A**), and the bottom of the membrane was blotted for GAPDH (**B**). The bottom section of the membrane was subsequently stripped and reblotted for DBP (**C**).

**Additional file 3.**

**Additional file 4.**

**Additional file 5.**

**Additional file 6.**

## Data Availability

All sequencing data have been submitted to NCBI GEO and are available via accession number GSE158655.

## Abbreviations

BMALI: Brain and Muscle Arnt-like protein-1
DBP: D Box Binding Protein
EDTA: Ethylenediamine tetraacetic acid
GAPDH: Glyceraldehyde-3-phosphate dehydrogenase
Per2: Period 2
QPCR: Quantitative polymerase chain reaction
