# Supplementary figures and images for: Methamphetamine-induced changes in myocardial gene transcription are sex-dependent
Source: BMC Genomics. 2021 Apr 12;22:259. doi: 10.1186/s12864-021-07561-x (PMC8042975; doi:10.1186/s12864-021-07561-x)

A

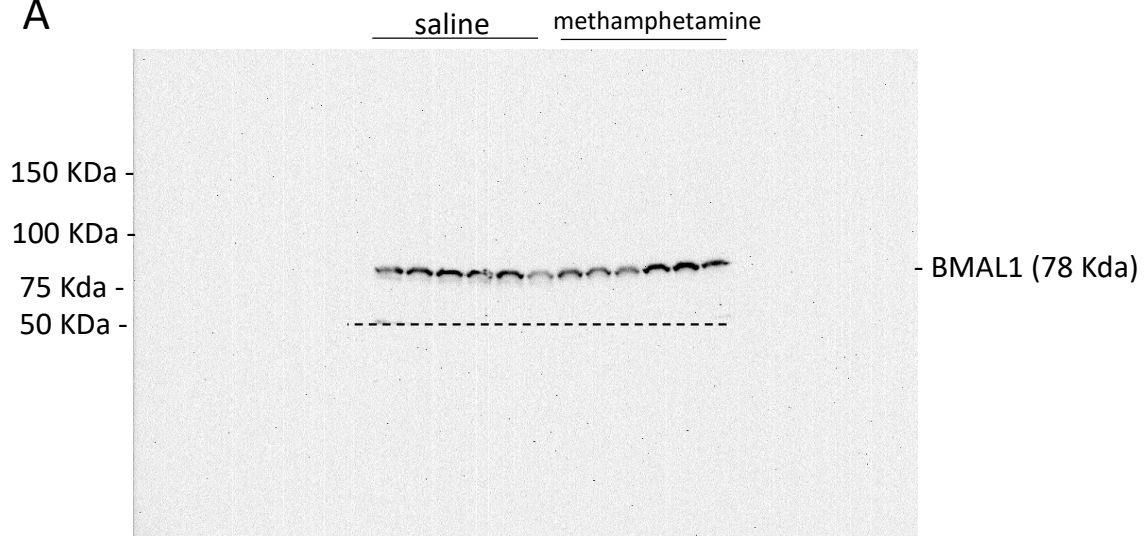

B

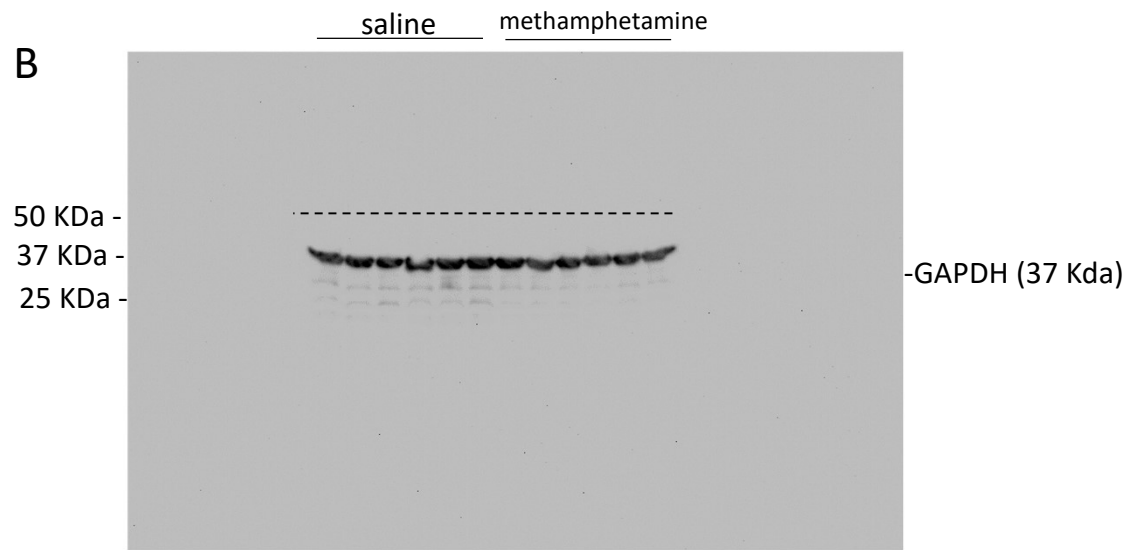

C

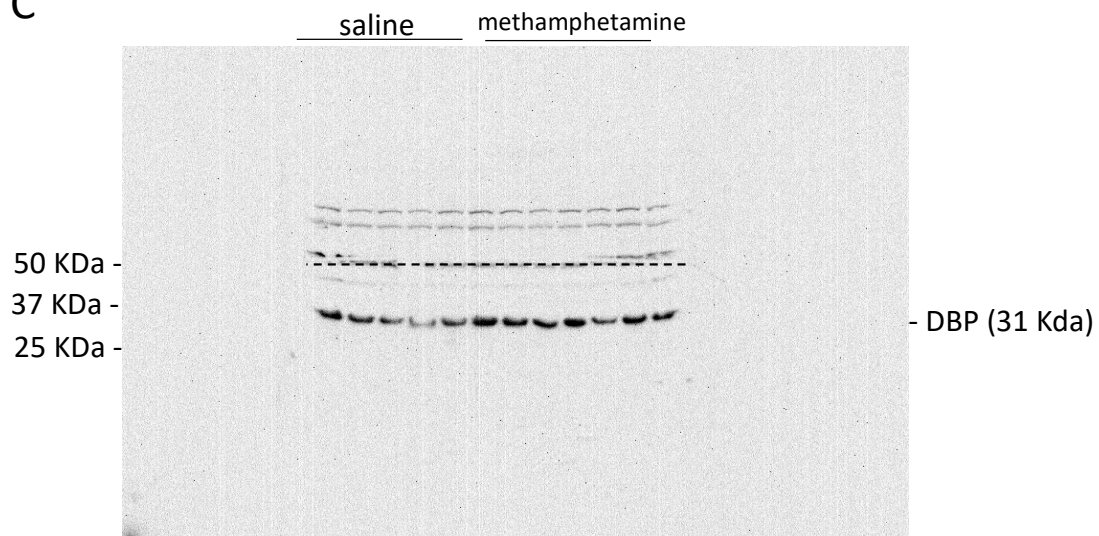

Supplement: Supplementary file 2 — Additional file 2: Supplemental Figure 2. Full length western blots for BMAL1, DBP, and GAPDH. Proteins from saline and methamphetamine-treated rat hearts were separated on a 10% polyacrylamide gel and blotted onto nitrocellulose membrane. The membrane was cut at the 50 KDa marker (indicated by the dotted line). The top part of the membrane was blotted for BMAL1 (A), and the bottom of the membrane was blotted for GAPDH (B). The bottom section of the membrane was subsequently stripped and reblotted for DBP (C). [file 12864_2021_7561_MOESM2_ESM.pdf]
